# Alignment Between Large Language Models and Consensus in Endoscopic Spinal Disc Surgery: A Comparative Analysis Against the Neurocore‐SENSED

**DOI:** 10.1002/jsp2.70216

**Published:** 2026-09-04

**Authors:** Abdullah İyigün, Göker Yurdakul, Alper Dünki, Mehmet Ali Yayla

**Affiliations:** ^1^ Clinic of Orthopaedics and Traumatology Ümraniye Training & Research Hospital Ümraniye Türkiye; ^2^ Department of Orthopaedics and Traumatology Bozok University Yozgat Merkez Türkiye

**Keywords:** artificial intelligence, ChatGPT, Claude, Delphi consensus, endoscopic spine surgery, Gemini, large language model

## Abstract

**Background:**

The Neurocore‐SENSED framework, derived from a three‐round modified Delphi process involving 77 international spine surgeons, provides a structured reference standard for reporting in endoscopic spine surgery (ESS) for disc disease. The ability of large language models (LLMs) to reproduce graded levels of expert agreement within a reporting framework has not been examined in ESS.

**Objective:**

To evaluate the extent to which three contemporary LLMs align with the Neurocore‐SENSED consensus and whether they discriminate between items of differing consensus level.

**Methods:**

In January 2026, each of the 166 framework items with published item‐level endorsement data was submitted once to GPT‐5.2, Gemini 3 Pro, and Claude Sonnet 4.6, in independent sessions with web retrieval disabled. Models selected one of five ordered response options directly. Alignment was assessed by Spearman correlation with panel endorsement and by four measures of categorical agreement, each with bootstrap 95% confidence intervals. Separate protocols examined response stability, response format, and prior exposure to the consensus.

**Results:**

All three models correlated significantly with panel endorsement. Gemini 3 Pro aligned most closely (*r*_*s*_ = 0.709, 95% CI 0.627–0.776), exceeding Claude Sonnet 4.6 (*r*_*s*_ = 0.622; *p* = 0.009) and GPT‐5.2 (*r*_*s*_ = 0.565; *p* < 0.001), which did not differ. Categorical agreement was limited and equivalent across models, with observed agreement of 0.506–0.518 and overlapping confidence intervals for all marginal‐robust statistics. Between 81.3% and 95.8% of responses fell in the top two categories, and discordance was almost entirely unidirectional: 31 items were endorsed by every model but by fewer than half the panel, against one item in the opposite direction. Weighted kappa was not interpretable in this setting.

**Conclusion:**

Current LLMs reproduce the broad rank ordering of expert endorsement in ESS reporting but cannot reliably distinguish endorsed from contested propositions, principally because of a positive response bias.

## 1 Introduction

Endoscopic spine surgery (ESS) has undergone remarkable evolution over recent decades, progressing from early percutaneous nucleotomy techniques to minimally invasive procedures capable of addressing a wide spectrum of spinal pathologies across cervical, thoracic, and lumbar regions [1, 2]. The increasing adoption of ESS in contemporary spine care has been driven by its demonstrated advantages, which include reduced tissue trauma, lower infection rates, diminished blood loss, shorter hospital stays, and accelerated return to daily activities [3, 4]. Nonetheless, the field continues to suffer from substantial heterogeneity in patient selection criteria, operative technique reporting, and outcome measurement, a limitation that impedes multicenter collaboration and the development of comprehensive evidence‐based practice guidelines [5].

To address this gap, Al Barajraji et al. recently conducted the Neurocore‐SENSED consensus study, an open and decentralized three‐round modified Delphi process involving 77 international spine surgeons [6]. This initiative produced a core outcome set and reporting guidelines for ESS applied to disc disease, establishing agreement on 99 of 183 proposed items spanning patient characteristics, clinical and surgical practices, and outcomes. The resulting framework provides a structured and internationally endorsed reference standard for ESS reporting. As with any Delphi‐derived framework, it records the aggregated judgment of its participating panel rather than an externally validated criterion of clinical correctness, a distinction that conditions the interpretation of any comparison made against it.

Large language models (LLMs), artificial intelligence systems trained on large bodies of text including the scientific literature, have become widely used tools in medical research [7, 8]. Systems such as OpenAI GPT, Google Gemini, and Anthropic Claude have demonstrated strong performance across a range of medical tasks, including answering board examination questions, synthesizing clinical guidelines, and supporting diagnostic reasoning [9, 10]. Their capacity to process complex clinical queries has prompted interest in whether they can represent, or approximate, collective expert judgment.

The extent to which LLMs can replicate the collective judgment of surgical specialists nevertheless remains poorly characterized. Prior evaluations of artificial intelligence in spine surgery have largely addressed patient education tasks rather than the appraisal of clinical parameters that characterizes Delphi‐based consensus methodology [11, 12]. Whether model responses align with expert consensus, and whether they can distinguish between differing levels of agreement, has not been systematically examined in ESS.

This study addresses that gap by comparing the responses of three widely used LLMs with the 166 items of the Neurocore‐SENSED framework for which item‐level endorsement data are published. Using rank correlation together with several measures of categorical agreement, we assess how closely model responses track expert endorsement, whether they discriminate between consensus levels, and which thematic domains are most and least well aligned. The aim is to characterize the boundaries of these systems as representations of expert opinion, not to evaluate them as substitutes for expert judgment.

## 2 Materials and Methods

### 2.1 Study Design and Reference Standard

**FIGURE 1:**
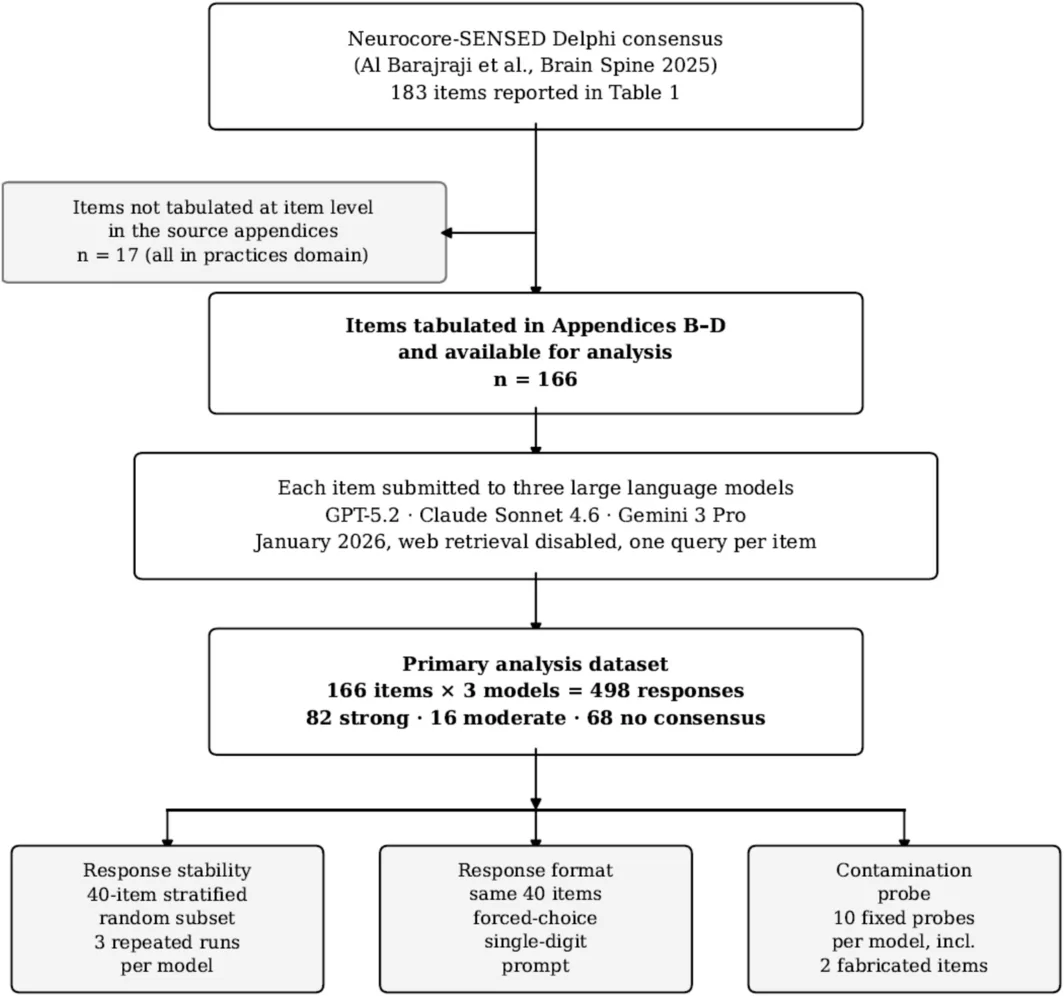
Derivation of the analyzed item set and study workflow. *Note:* Item‐level endorsement values are published for 166 of the 183 items reported in the source consensus framework. The three protocols shown at the foot of the figure were conducted and are described in Supplement S1, Section S1.3.

This was a cross‐sectional comparative study evaluating the agreement between responses generated by LLMs and an established international expert consensus. The reference standard was the Neurocore‐SENSED consensus framework, developed between January and April 2025 through a three‐round, web‐based modified Delphi process involving 77 spine surgeons from Europe (61%), Asia (14%), and North America (13%), of whom 58 completed all three rounds [6]. The consensus panel comprised spine neurosurgeons (32%), spine orthopedists (31%), general neurosurgeons (30%), and general orthopedists (7%). Propositions endorsed by at least 75% of participants were classified as strong agreement; those endorsed by 50%–75% were reconsidered in a subsequent round and required at least 66% for classification as moderate agreement; propositions falling below this threshold were classified as not reaching consensus. The classification applied to each item in the present analysis is the classification published by the consensus authors. It should be emphasized that this framework constitutes a reference standard of expert opinion rather than an external criterion of clinical truth. Delphi methodology aggregates the collective judgment of a defined panel and is therefore conditioned on that panel's geographic, specialty, and practice‐pattern composition. Agreement between a model and the consensus was accordingly interpreted as concordance with a documented expert position, not as clinical correctness, and divergence from consensus was not treated as evidence of error. The source publication reports a full item bank of 183 items across three domains: patient characteristics (65 items), practices (86 items), and outcomes (32 items). Item‐level endorsement percentages and consensus classifications are tabulated in Appendices B–D of that publication for 166 of these items; the 17‐item difference lies entirely within the practices domain and reflects the extent of item‐level tabulation in the source publication. All 166 items for which item‐level data were published were analyzed, without exclusion (Figure 1). Their distribution by domain and consensus level is shown in Table 1.

**TABLE 1:** Distribution of the 166 analyzed items by domain and Delphi consensus level.

Domain	Items analyzed	Strong agreement (≥ 75%)	Moderate agreement (66%–74%)	No consensus (< 66%)
Patient characteristics	65	30	7	28
Practice	69	32	7	30
Outcomes	32	20	2	10
Total	166	82	16	68

*Note:* Consensus categories follow the classification applied by Al Barajraji et al. Items are those tabulated in Appendices B–D of the source publication.

### 2.2 AI Model Selection and Query Administration

Three commercially available LLMs were evaluated: OpenAI GPT‐5.2, Google Gemini 3 Pro, and Anthropic Claude Sonnet 4.6. These were the general‐purpose models offered by each provider at the time of data collection, selected for their widespread availability, documented performance on medical knowledge benchmarks, and relevance to research applications. All queries were administered in January 2026 through each provider's standard consumer conversational interface. Each of the 166 items was submitted independently and once per model, in a new session with no prior conversation history, with web retrieval disabled and no system prompts or custom instructions applied. Where an item was nested beneath a parent item in the source questionnaire, the parent text was included so that the model saw the same context as the panelists. Full query conditions and the verbatim prompt are given in Supplement S1, Section S1.1.

### 2.3 Response Scoring

The prompt required each model to select exactly one of five ordered response options, anchored at strongly disagree and strongly agree, indicating the degree to which the model endorsed systematic reporting of the item in research on ESS for disc disease. Responses were therefore generated as discrete categorical selections by the models themselves and were transcribed to their corresponding numeric values (1–5) without interpretation. No human rating, coding, or conversion of free‐text output was involved at any stage, and inter‐rater reliability is consequently not applicable to this design. The response label emitted by each model and its corresponding ordinal value are reported for every item in Data S1.

### 2.4 Statistical Analysis

Spearman rank correlation coefficients (*r*_*s*_) were computed between the model response (1–5) and both the continuous panel endorsement percentage and the three‐level consensus classification, overall and stratified by consensus level and theme, with bootstrap 95% confidence intervals. Correlations between models were compared using the Williams modification of the Steiger test for dependent correlations, and theme‐level analyses were corrected by the Benjamini–Hochberg procedure. Categorical agreement was quantified after collapsing model responses into three categories (1–2, 3, 4–5). Because the response distributions were markedly skewed, observed agreement, linear weighted kappa, the prevalence‐adjusted bias‐adjusted kappa and Gwet's AC2 were reported together, the latter two being robust to marginal imbalance; kappa was interpreted using the benchmarks of Landis and Koch [13]. Sensitivity to the collapsing rule, to item hierarchy, and to the exclusion of items implicated in the exposure probe was assessed as described in Supplement S1, Section S1.2. Three additional protocols addressed response stability, response format, and prior exposure to the reference consensus (Supplement S1, Section S1.3). Significance was set at *p* < 0.05 (two‐tailed); analyses used Python 3.11 (SciPy 1.11).

### 2.5 TRIPOD‐LLM Adherence Statement

This study is reported in accordance with the TRIPOD‐LLM reporting guideline for studies using LLMs [14]. The applicable modules are those for evaluation of an existing model without development, tuning, or deployment; items relating to model development, training data curation, fine‐tuning, calibration, and clinical implementation are not applicable to this design. A completed checklist indicating where each applicable item is reported is provided as Appendix S1.

## 3 Results

### 3.1 Rank‐Order Alignment With the Consensus

All three models showed statistically significant positive rank‐order correlations with panel endorsement across the 166 items (Table 2). Gemini 3 Pro showed the strongest association (*r*_*s*_ = 0.709, 95% CI 0.627–0.776), followed by Claude Sonnet 4.6 (*r*_*s*_ = 0.622, 95% CI 0.512–0.715) and GPT‐5.2 (*r*_*s*_ = 0.565, 95% CI 0.452–0.659), all *p* < 0.001. Pairwise comparison of these dependent correlations showed that Gemini 3 Pro aligned more closely with the consensus than either Claude Sonnet 4.6 (*t* = 2.63, *p* = 0.009) or GPT‐5.2 (t = 3.36, *p* < 0.001), whereas Claude Sonnet 4.6 and GPT‐5.2 did not differ (*t* = 1.05, *p* = 0.296).

**TABLE 2:** Agreement between model responses and the Neurocore‐SENSED consensus (*n* = 166 items).

Model	Spearman *r*_*s*_ (95% CI)	*p*	Observed agreement	Linear weighted *κ* (95% CI)	PABAK	Gwet's AC2 (95% CI)	Responses at 4–5 (%)
Claude Sonnet 4.6	0.622 (0.512–0.715)	< 0.001	0.506	0.191 (0.124–0.260)	0.012	0.356 (0.205–0.495)	81.3
Gemini 3 Pro	0.709 (0.627–0.776)	< 0.001	0.512	0.152 (0.094–0.218)	0.024	0.355 (0.205–0.492)	87.3
GPT‐5.2	0.565 (0.452–0.659)	< 0.001	0.518	0.076 (0.024–0.140)	0.036	0.344 (0.202–0.483)	95.8

*Note:* Spearman correlations are between the model score (1–5) and the panel endorsement percentage. Weighted *κ*, PABAK, and AC2 compare the model score, mapped to three categories (1–2/3/4–5), with the three consensus levels. Confidence intervals are bootstrap percentile intervals (5000 resamples). Observed agreement is near‐identical across models while weighted *κ* varies more than twofold, and AC2 shows overlapping confidence intervals for all three models; *κ* in this setting reflects the skew of the response distribution rather than differential agreement.

### 3.2 Categorical Agreement and the Limits of Kappa

Absolute categorical agreement with the three‐level classification was limited for all models. Observed agreement was closely similar across models (0.506, 0.512, and 0.518 for Claude Sonnet 4.6, Gemini 3 Pro and GPT‐5.2 respectively), yet linear weighted kappa varied more than twofold across the same models (0.191, 0.152, and 0.076). This divergence is a property of the statistic rather than of the models: kappa penalizes the marginal skew of the response distribution, which differed substantially between models. Gwet's AC2, which is robust to marginal imbalance, was effectively identical for all three (0.356, 0.355, and 0.344) with fully overlapping confidence intervals (Table 2; full cross‐tabulations in Table S1). Under alternative collapsing rules the ordering of models by weighted kappa reversed (Table 3). Weighted kappa is therefore not interpretable as a measure of differential model performance in this setting, and no ranking of models by categorical agreement is supported by these data.

**TABLE 3:** Sensitivity analyses.

Analysis	Claude Sonnet 4.6	Gemini 3 Pro	GPT‐5.2	Statistic
Score‐to‐category mapping: 1–2/3/4–5 (primary)	0.191	0.152	0.076	*κ*_w_
Score‐to‐category mapping: 1–3/4/5	0.450	0.496	0.312	*κ*_w_
Score‐to‐category mapping: 1–2/3–4/5	0.292	0.373	0.290	*κ*_w_
Item hierarchy: Parent items only (*n* = 128)	0.612	0.694	0.534	*r*_*s*_
Item hierarchy: Sub‐items only (*n* = 38)	0.620	0.743	0.622	*r*_*s*_
Excluding the item recalled in the contamination probe (*n* = 165)	0.643	0.722	0.564	*r*_*s*_

*Note:* The ordering of models by weighted κ reverses under alternative score‐to‐category mappings, whereas the rank‐order correlation is stable across item‐hierarchy strata and after removing the single item for which a model reproduced the published endorsement percentage in the contamination probe.

### 3.3 Response Distribution and Ceiling Effect

The response distributions of all three models were markedly right‐skewed, with 81.3%, 87.3%, and 95.8% of responses at the top two categories for Claude Sonnet 4.6, Gemini 3 Pro and GPT‐5.2 respectively. The effect was most pronounced in the complications theme, where Claude Sonnet 4.6 and Gemini 3 Pro assigned the maximum response to all 14 items, including items endorsed by only 42% and 48% of the panel, leaving no variance from which a correlation could be computed (Table 4). Discordance was strongly asymmetric in direction. In 31 items the panel endorsement fell below 50% while all three models responded in the agreement range, whereas only a single item was endorsed by at least 85% of the panel without corresponding endorsement by every model. Representative examples in the technical practice theme included the use of forceps and graspers (30% endorsement), the use of cannulas and tubes (43%), device speed settings (38%), and regulatory approval details (35%), each of which all three models endorsed for systematic reporting (Supplementary Table S2). Under a forced‐choice format the proportion of responses in the top two categories fell by 15.0 percentage points for Gemini 3 Pro and 30.0 points for GPT‐5.2 (both *p* < 0.001), but by only 2.5 points for Claude Sonnet 4.6 (*p* = 0.317), and the three models converged (Table 5). The magnitude of the ceiling effect, and the differences between models in that magnitude, are therefore partly attributable to the elicitation format rather than to the models alone; rank‐order correlations were nevertheless preserved under the forced‐choice condition.

**TABLE 4:** Theme‐level correlations between model scores and panel endorsement.

Theme	*n*	Claude Sonnet 4.6	Gemini 3 Pro	GPT‐5.2
In clinical practice, what should be systematically reported?	17	0.741*	0.902*	0.819*
In surgical practice, what should be systematically reported?	29	0.886*	0.868*	0.595*
In technical practice, what should be systematically reported?	23	0.306	0.591*	0.187
What should be the reported indicators?	16	0.841*	0.812*	0.605*
Which active treatments should be systematically reported?	11	0.493	0.848*	0.324
Which clinical severity scoring system should be systematically used?	12	0.771*	0.738*	0.681*
Which comorbidities should be systematically reported?	11	0.702*	0.785*	0.750*
Which complications should be reported?	14	—	—	0.034
Which demographic data should be systematically reported?	13	0.578*	0.728*	0.654*
Which initial patient complaint should be systematically reported?	8	0.760*	0.760*	0.760*
Which morphological severity criteria should be systematically reported?	8	0.694	0.643	0.764*

*Note:* Values are Spearman *r*_*s*_; asterisks denote *p* < 0.05 after Benjamini–Hochberg correction, which 24 of 31 evaluable correlations survived. Three themes with fewer than four items were not analyzed. For the complications theme, Claude Sonnet 4.6 and Gemini 3 Pro assigned the maximum score to all 14 items, including items endorsed by only 42% and 48% of the panel, so no correlation could be computed; GPT‐5.2 varied on a single item.

**TABLE 5:** Response stability and effect of response format (40‐item stratified random subset).

Model	Krippendorff's *α*	Identical across 3 runs (%)	Range ≤ 1 point (%)	Responses at 4–5, free text (%)	Responses at 4–5, forced choice (%)	Difference (pp)	*p*
Claude Sonnet 4.6	0.865	67.5	85.0	75.0	72.5	−2.5	0.317
Gemini 3 Pro	0.932	55.0	90.0	85.0	70.0	−15.0	< 0.001
GPT‐5.2	0.942	70.0	90.0	95.0	65.0	−30.0	< 0.001

*Note:* Each item was submitted three times per model in independent sessions. The forced‐choice condition constrained the model to return a single digit. *p*‐values are from Wilcoxon signed‐rank tests on paired item‐level scores. The forced‐choice prompt differed from the original prompt in response format and additionally instructed the model not to decline to answer; the two changes cannot be separated in this design.

### 3.4 Subgroup and Theme‐Level Analysis

Stratified by consensus level, all models correlated most strongly with endorsement within the strong‐agreement stratum (*n* = 82; *r*_*s*_ = 0.463, 0.398, and 0.350 for Gemini 3 Pro, Claude Sonnet 4.6, and GPT‐5.2). Correlations within the no‐consensus stratum (*n* = 68) were attenuated but significant (*r*_*s*_ = 0.373, 0.288, and 0.292). No model reached significance in the moderate stratum, which comprised 16 items; this stratum is small and results from it should be regarded as exploratory.

Across the 14 themes represented by four or more items, 24 of 31 evaluable correlations remained significant after Benjamini–Hochberg correction (Table 4). Alignment was strongest in clinical and surgical practice reporting. In the technical practice theme, Gemini 3 Pro alone showed significant alignment (*r*_*s*_ = 0.591, corrected *p* < 0.05), whereas Claude Sonnet 4.6 (*r*_*s*_ = 0.306) and GPT‐5.2 (*r*_*s*_ = 0.187) did not, indicating that reduced discrimination in technical domains is model‐dependent rather than general.

### 3.5 Prior Exposure to the Reference Consensus

With web retrieval disabled, no model reproduced the publication details, panel size, or authorship of the reference consensus, and none reported the item‐level endorsement percentages reliably (Table 6). GPT‐5.2 returned confident numerical answers to seven probes, of which one was correct; the same model returned an identical value for a fabricated item, indicating a default estimate rather than retrieval of stored content. Gemini 3 Pro produced one incorrect endorsement percentage and one incorrect consensus classification, and Claude Sonnet 4.6 declined to answer all item‐level probes. Excluding the single item for which a correct value was produced changed the corresponding correlation by less than 0.002 (Table 3).

**TABLE 6:** Training‐data contamination probe.

Probe	Content requested	Claude Sonnet 4.6	Gemini 3 Pro	GPT‐5.2
P01	Publication details (year, journal, panel size)	No recall	No recall	No recall
P02	Lead and senior authors	No recall	No recall	No recall
P03	Number of items assessed and agreed	No recall	No recall	Confabulated
P04	Endorsement thresholds used	No recall	Partial	Partial
P05	Endorsement % for tobacco consumption	No recall	No recall	Confabulated
P06	Consensus status of wrong‐site surgery	No recall	Confabulated	Confabulated
P07	Endorsement % for irrigation pump settings	Partial	Confabulated	Accurate recall
P08	Endorsement % for a fabricated item	No recall	No recall	Confabulated
P09	Classification of a fabricated item	No recall	No recall	Confabulated
P10	Retrieval check	No retrieval used	No retrieval used	No retrieval used

*Note:* Probes P08 and P09 name items that do not exist in the Neurocore‐SENSED framework and serve as controls. GPT‐5.2 returned a confident endorsement percentage for both, including the same value (92%) that it gave for the one real item it recalled correctly (P07), indicating that this value represents a default estimate rather than retrieval of stored content. Removing that item from the primary analysis changed the GPT‐5.2 correlation by less than 0.002 (Table 3). No model reproduced publication details or item‐level endorsement values reliably. P10 is a retrieval check rather than a recall probe; all three models stated that no external source was consulted.

## 4 Discussion

This study evaluated the concordance between three LLMs and the Neurocore‐SENSED expert consensus for ESS in disc disease. All three models showed statistically significant positive rank‐order correlations with the Delphi‐derived consensus, yet none could reliably assign items to the correct consensus tier. The findings indicate that current models reproduce the broad contour of expert opinion while lacking the resolution to distinguish endorsed from contested propositions, with a systematic positive response bias constituting the principal constraint.

A conceptual constraint intrinsic to this design merits explicit statement. The Neurocore‐SENSED consensus, like all Delphi products, is a socially constructed reference standard: it captures what a defined panel agreed should be reported, not what has been demonstrated to influence outcome. The panel comprised 77 surgeons drawn predominantly from Europe (61%), with limited representation from Asia (14%) and North America (13%) and none reported from Latin America, Africa, or the Middle East; spine neurosurgeons and spine orthopedists together accounted for 63% of participants. Because indication thresholds and instrumentation preferences differ between regions and specialties, item endorsements are plausibly shaped by panel composition. Two consequences follow. A model response that diverges from the consensus is not thereby incorrect; it may reflect a practice pattern prevalent in the global literature but underrepresented in the panel. Conversely, concordance may partly reflect shared derivation from the same predominantly Western, English‐language evidence base rather than independent convergence on clinically valid positions. The coefficients reported here therefore measure alignment with a particular expert position at a particular moment, not clinical validity.

### 4.1 Rank‐Order Alignment and the Limits of Categorical Agreement

The moderate‐to‐strong Spearman correlations confirm that the rank ordering of item endorsement by these models is broadly consistent with that of the expert panel, and this is consistent with prior evaluations of language models against clinical guidelines in spine surgery. Khoylyan et al. reported that GPT‐4 aligned with NASS evidence‐based guidelines for lumbar fusion in 88.2% of cases [15], and Chester and Mandler found reasonable correlation between ChatGPT and expert consensus statements on surgical site infection prevention in pediatric spine surgery, particularly for binary endorsement tasks [16]. The present findings extend this literature from diagnosis and management to the more granular level of outcome reporting.

Rank correlation and categorical agreement, however, measure different constructs: a model may order items correctly while still failing to place them in the correct tier. The weighted kappa values obtained here all fell within the slight range by the Landis and Koch criteria [13]. It is important not to over‐read the differences between them. Observed agreement was closely similar across models, and Gwet's AC2, which does not penalize marginal imbalance, was effectively identical, with fully overlapping confidence intervals. The apparent spread in weighted kappa therefore reflects how far each model's response distribution departed from the marginal distribution of the consensus classification, not a difference in how well each model agreed with it, and the ordering reverses under alternative collapsing rules. No ranking of these models by categorical agreement is supported by the data. The substantive conclusion is common to all three: current models replicate the broad contour of expert opinion but cannot reliably distinguish items experts endorsed from those over which meaningful disagreement persists.

### 4.2 The Positive Response Bias

The most consequential finding is the systematic positive response bias. Between 81% and 96% of responses fell in the top two categories irrespective of the consensus level of the item, and in the complications theme two of the three models assigned the maximum response to every item, including propositions endorsed by fewer than half the panel. This ceiling effect is not confined to ESS: Chundi et al., evaluating language models in orthopedic surgery, observed that at least 95% of responses returned top confidence scores, a pattern attributed to training that prioritizes fluency over calibration [10]. The bias appears to reflect properties intrinsic to general‐purpose training. Models exposed to large volumes of published recommendations will assign high endorsement to almost any clinically plausible proposition, a form of anchoring to the most probable response rather than genuine discrimination. Saturno et al. observed an analogous pattern, finding that ChatGPT‐4 aligned more strongly with lower‐quality evidence than with Level I recommendations in cervical spine injury management, suggesting that models reflect the volume of literature supporting a claim rather than its evidentiary grade [17].

The elicitation format contributes materially to the magnitude of this effect. When the same items were re‐administered in a forced‐choice format, the proportion of top‐category responses fell substantially for two of the three models and the models converged, indicating that between‐model differences in apparent bias are partly an artifact of how the response was elicited rather than a stable property of the models. Rank‐order correlations were preserved under this condition. The bias is therefore real but its measured magnitude, and particularly any comparison of magnitude between models, is protocol‐dependent.

### 4.3 Domain‐Level Heterogeneity

Alignment was not homogeneous across themes. Correlations were strongest and most consistent in the clinical and surgical practice themes and weakest in technical practice, though not uniformly so: one model retained significant alignment in that theme while the other two did not, indicating that reduced discrimination in technical domains is model‐dependent rather than a general property. This pattern is consistent with Almekkawi et al., who found that ChatGPT and Claude described imaging findings and general treatment options in detail but diverged from expert surgeons when selecting among specific technical approaches [18]. Technical parameters in ESS (irrigation specifications, burr types, endoscope configurations) demand familiarity with detail that is less represented in the literature than the clinical and outcome domains forming the core of published guidelines. Saylan et al., comparing language models with experienced spinal surgeons on controversial management scenarios, similarly found low agreement and a consistent failure to account for patient‐specific factors [19]. Comparable observations have been reported outside spine surgery in risk‐sensitive imaging contexts, where fluent textual performance did not translate into reliable technical accuracy [20, 21].

### 4.4 Inter‐Model Differences and Comparison With the Consensus

One model achieved significantly higher rank‐order correlation than the other two, which did not differ from each other; no model was superior on every metric. These variations plausibly reflect differences in training data composition, architecture, and the extent of medical fine‐tuning. Chundi et al. likewise reported substantial variability across models in orthopedics, with different systems showing distinct patterns of strength across subspecialty domains [10], and Liu et al. observed that artificial intelligence can match human quality in general clinical scenarios while falling short in technically detailed fields [22]. The absence of a single dominant model reinforces the view that performance is domain‐specific and that benchmarks from one field do not generalize. The reference standard itself reached agreement on only 99 of 183 items, reflecting substantial heterogeneity among international experts [6]. When evaluated against this standard, it is notable that the models separated high‐consensus items, such as radicular symptoms and surgical indications, from less consistently endorsed technical parameters. They encode a broad understanding of which categories are considered clinically important without reliably distinguishing endorsed from non‐endorsed items within those categories. Because model architectures are revised frequently, these findings characterize the systems as deployed in January 2026 and warrant longitudinal reassessment.

## 5 Limitations

Several limitations should be acknowledged. First, the findings establish concordance with a documented expert consensus rather than clinical validity. The reference standard is an expert‐opinion construct rather than an objective clinical criterion, and its panel was geographically and professionally non‐uniform; items not reaching consensus may represent genuine equipoise or regional divergence rather than positions a model should have scored low. No claim about the safety or quality of model‐generated surgical reasoning can be drawn from these data. Second, model responses were elicited at a single point in time and each item was queried once in the primary dataset; a repeat‐sampling protocol on a stratified subset showed high but not perfect stability, so a small proportion of individual item scores would differ on re‐querying. Third, the collapsing of five ordered response options onto a three‐level classification is one of several defensible choices; the rank‐order findings were robust across alternative mappings, whereas the categorical statistics were not, which is why no model comparison is drawn from them. Fourth, all models exhibited a pronounced ceiling effect whose magnitude is partly attributable to the elicitation format, and the forced‐choice comparison altered both response format and instruction simultaneously, so these influences cannot be separated in this design. Fifth, weighted kappa is sensitive to marginal distribution, and the low values obtained reflect distributional mismatch as much as substantive disagreement; this motivated the additional marginal‐robust statistics reported here. Sixth, item‐level data were available for 166 of the 183 items in the source framework, and the 17 items without published item‐level values fall disproportionately in the non‐consensus stratum, which may attenuate estimates of discrimination in that stratum. Finally, the analysis was restricted to three commercially available general‐purpose models queried without retrieval augmentation, chain‐of‐thought prompting, or fine‐tuning; whether these techniques would improve discrimination, and whether the findings generalize to other systems, remains to be established.

## 6 Conclusion

Current LLMs demonstrate meaningful rank‐order alignment with an international expert consensus on reporting standards for ESS, but do not reliably discriminate between items of differing consensus level, principally because of a systematic positive response bias whose magnitude depends in part on how responses are elicited. These findings delineate both the potential and the present boundaries of language models as a surrogate for expert consensus in the standardization of surgical practice. Such models can help investigators orient themselves within the broad structure of reporting in ESS; on the present evidence, they cannot substitute for formal consensus processes where the distinction between endorsed and contested propositions is what matters.

## Author Contributions

**Abdullah İyigün:** methodology, writing – original draft, conceptualization, formal analysis, supervision. **Alper Dünki:** writing – review and editing, writing – original draft. **Göker Yurdakul:** data curation, software. **Mehmet Ali Yayla:** writing – original draft, project administration, data curation.

## Funding

The authors have nothing to report.

## Ethics Statement

This study did not involve human participants, patient data, or identifiable information. It analyzed publicly available item‐level data from a published consensus framework together with responses generated by commercially available language models. Institutional review board approval and informed consent were therefore not required.

## Conflicts of Interest

The authors declare no conflicts of interest.

## Supporting information


**Data S1:** Complete datasets.

**Supplement S1:** Prompts and analytical protocols.

**Appendix S1:** TRIPOD‐LLM checklist.

**Table S1:** Cross‐tabulation of consensus level against collapsed model response (*n* = 166 items).
**Table S2:** Items showing the greatest discordance between panel endorsement and model response.

## Data Availability

The data supporting the findings of this study are available as Supporting Information. Data S1 contains the complete 166‐item score matrix, including the response label emitted by each model and its corresponding ordinal value, together with the repeated‐sampling and forced‐choice datasets, the cross‐tabulations, and the verbatim responses to the contamination probe. The verbatim prompts and the full analytical protocols are provided in Supplement S1. No patient‐level or otherwise identifiable data were generated or analyzed.
