# Supplementary material for: Alignment Between Large Language Models and Consensus in Endoscopic Spinal Disc Surgery: A Comparative Analysis Against the Neurocore‐SENSED
Source: JOR Spine. 2026 Sep 4;9(3):e70216. doi: 10.1002/jsp2.70216 (PMC13545091; doi:10.1002/jsp2.70216)
Supplement: Supplementary file 1 [file JSP2-9-e70216-s004.pdf]

TRIPOD+LLM Checklist — Completed

Manuscript: Alignment Between Large Language Models and Consensus in Endoscopic Spinal Disc Surgery: A Comparative Analysis Against the Neurocore-SENSED
Research design: E (evaluation of existing LLMs; no model development, tuning, or clinical deployment)
LLM task: Classification / question answering (single ordered-category selection per item)
Page numbers refer to the manuscript file as paginated in the submitted Word document. "NA" = item not applicable to this research design or LLM task per the TRIPOD+LLM design column.

Section / Topic	Item	Checklist Item	Design / Task	Reported on Page
**Abstract**				
**Title**	**2a**	Identify the study as developing, fine-tuning, and/or evaluating the performance of an LLM, specifying the task, the target population, and the outcome to be predicted.	All / All	p. 1 (Title)
**Abstract**	**2b**	Provide a brief explanation of the healthcare context, use case and rationale for developing or evaluating the performance of an LLM.	E,H / All	p. 1 (Abstract, Background)
**Objectives**	**2c**	Specify the study objectives, including whether the study describes LLM development, tuning, and/or evaluation.	All / All	p. 1 (Abstract, Objective)
**Methods**	**2d**	Describe the key elements of the study setting.	All / All	p. 1 (Abstract, Methods)
	**2e**	Detail all data used in the study, specify data splits and any selective use of data.	M,D,E / All	p. 1 (Abstract, Methods — 166 consensus items with published item-level endorsement data; evaluation only, therefore no training/tuning splits)
	**2f**	Specify the name and version of LLM used.	All / All	p. 1 (Abstract, Methods — GPT-5.2, Gemini 3 Pro, Claude Sonnet 4.6)
	**2g**	Briefly summarize the LLM-building steps (fine-tuning, reward modeling, RLHF, etc.).	M,D / All	NA — Not Required (evaluation-only design)
	**2h**	Describe the specific tasks performed by the LLMs, highlighting key inputs and outputs used in the final LLM.	All / All	p. 1 (Abstract, Methods)
	**2i**	Specify the evaluation datasets/populations used, including the endpoint evaluated, whether held out during training/tuning, and the measures used to evaluate LLM performance.	All / All	p. 1 (Abstract, Methods)
**Results**	**2j**	Give an overall report and interpretation of the main results.	All / All	pp. 1–2 (Abstract, Results)
**Discussion**	**2k**	Explicitly state any broader implications or concerns that have arisen in light of these results.	All / All	p. 2 (Abstract, Conclusion)
**Other**	**2l**	Give the registration number and name of the registry or repository (if relevant).	H / All	NA — Not Required
**Introduction**				
**Background**	**3a**	Explain the healthcare context / use case and rationale for developing or evaluating the LLM, including references to existing approaches and models.	All / All	pp. 2–3 (Introduction, paragraphs 1–4)
	**3b**	Describe the target population and the intended use of the LLM in the context of the care pathway, including its intended users.	E,H / All	p. 3 (Introduction, final paragraph); pp. 15–16 (Conclusion)
**Objectives**	**4**	Specify the study objectives, including whether the study describes initial development, fine-tuning, or validation of an LLM.	All / All	p. 3 (Introduction, final paragraph); p. 7 (TRIPOD-LLM adherence statement — evaluation only)
**Methods — Data**				
**Data**	**5a**	Describe the sources of data separately for the training, tuning, and/or evaluation datasets and the rationale for using these data.	All / All	pp. 3–4 (Study Design and Reference Standard); p. 5 (Table 1). Evaluation data only: published item-level endorsement values of the Neurocore-SENSED Delphi consensus
	**5b**	Describe the relevant data points and provide a quantitative and qualitative description of their distribution and other descriptors (source, languages, countries of origin).	All / All	p. 3 (panel composition, geography); pp. 4–5 (166 items, three domains); p. 5 (Table 1)
	**5c**	State the date of the oldest and newest item of text used in development and in the evaluation datasets.	M,D,E,H / All	p. 3 (consensus developed January–April 2025); p. 6 (queries administered January 2026). Model pre-

				training cut-off dates are not disclosed by the providers
	**5d**	Describe any data pre-processing and quality checking, including whether this was similar across corpora, institutions, and sociodemographic groups.	All / All	p. 4 (item classifications used exactly as published by the consensus authors; no re-derivation); p. 6 (Response Scoring — verbatim transcription of model selections, no interpretation)
	**5e**	Describe how missing and imbalanced data were handled and reasons for omitting any data.	M,D,E / All	p. 4 (17 of 183 items lack published item-level values; all 166 available items analyzed without exclusion; Figure 1); p. 15 (Limitation 6)
**Methods — Analytical Methods**				
**Analytical Methods**	**6a**	Report the LLM name, version, and last date of training or use during inference.	All / All	p. 6 (AI Model Selection and Query Administration — GPT-5.2, Gemini 3 Pro, Claude Sonnet 4.6; all queries January 2026)
	**6b**	Specify the LLM architecture and building steps, including hyperparameter tuning, prompt engineering, and inference settings.	M,D,E / All	p. 6 (standard consumer interfaces, new session per item, web retrieval disabled, no system prompts or custom instructions); Appendix S1.1 (verbatim prompt and full query conditions). Temperature/seed are not user-configurable in the consumer interfaces used
	**6c**	Report details of the LLM development process, training/fine-tuning procedures, alignment strategy and alignment goals.	M,D / All	NA — Not Required (proprietary models evaluated as deployed)
	**6d**	Specify the initial and post-processed output of the LLM.	All / All	p. 6 (Response Scoring — one of five ordered response options; transcribed to numeric values 1–5 without interpretation)
	**6e**	Provide details and rationale for any classification and how probabilities were determined and thresholds identified.	All / C,OF	Not Required. Partially addressed p. 6 (collapsing of 1–5 scores into three categories) and p. 8 (Table 3, sensitivity to the collapsing rule)
	**6f**	Include metrics capturing the quality of generative outputs (consistency, relevance, accuracy) compared to gold standards.	All / QA,IR,DG,SS, MT	Not Required. Response consistency nevertheless reported: p. 10 (Table 5, Krippendorff's α across three independent runs)
	**6g**	Report the outcome metrics' relevance to the downstream task at deployment time and correlation of the metric with human evaluation of the text.	E,H / All	p. 6 (Statistical Analysis — agreement with the expert panel is the metric); pp. 12–13 (Discussion — what the reference standard represents and the limits of categorical agreement)
**Methods — LLM Output**				
**LLM Output**	**7a**	Clearly define the outcome, how LLM predictions were calculated, and evaluation metrics.	E,H / All	p. 6 (Response Scoring; Statistical Analysis); pp. 6–7 (Spearman r⍰, observed agreement, linear weighted κ, PABAK, Gwet's AC2, bootstrap CIs)
	**7b**	If outcome assessment requires subjective interpretation, describe assessor qualifications, instructions, demographics, and inter-assessor agreement.	All / All	p. 6 (Response Scoring — models selected a category directly; no human rating, coding, or conversion of free text, therefore inter-rater reliability is not applicable)
	**7c**	Specify how performance was compared to other LLMs, humans, and other benchmarks or standards.	All / All	p. 6 (Statistical Analysis — Williams/Steiger test for dependent correlations; comparison against the Delphi panel); pp. 7–8 (Results, Table 2)
**Methods — Annotation**				
**Annotation**	**8a**	If annotation was done, report how text was labeled, including annotation guidelines with examples.	All / All	NA — no human annotation was performed (p. 6, Response Scoring)
	**8b**	If annotation was done, report how many annotators labeled the dataset(s) and the proportion double-annotated.	All / All	NA — no human annotation was performed (p. 6)
	**8c**	If annotation was done, provide the background and experience of annotators and inter-annotator agreement.	All / All	NA — no human annotation was performed (p. 6)
**Methods — Prompting, Compute, Ethics**				
**Prompting**	**9a**	If research involved prompting LLMs, provide details on prompt design, curation, and selection.	All / All	p. 6 (query administration and response scoring); Appendix S1.1 (verbatim prompt); p. 10 and Appendix S1.3 (forced-choice prompt variant)
	**9b**	If research involved prompting LLMs, report what data were used to develop the prompts.	All / All	p. 6 (item text taken verbatim from the Neurocore-SENSED questionnaire, with parent item text included for nested items); Appendix S1.1
**Summarization**	**10**	Describe any preprocessing of the data before summarization.	All / SS	NA — Not Required (no summarization task)
**Instruction Tuning / Alignment**	**11**	If instruction tuning/alignment strategies were used, describe the instructions, evaluation interface, and evaluator populations.	M,D / All	NA — Not Required (no tuning or alignment performed)
**Compute**	**12**	Report compute, or proxies thereof, required to carry	M,D,E / All	p. 6 (queries run through provider consumer

		out methods.		interfaces; no local training or inference compute used); analyses performed on a standard desktop workstation with Python 3.11 (p. 7)
**Ethics Approval**	**13**	Name the institutional research board or ethics committee that approved the study and describe informed consent or the waiver.	All / All	p. 16 (Ethics Statement — no human participants, patient data, or identifiable information; institutional review board approval and informed consent not required)
**Methods — Open Science**				
**Open Science**	**14a**	Give the source of funding and the role of the funders.	All / All	p. 16 (Funding Statement)
	**14b**	Declare any conflicts of interest and financial disclosures for all authors.	All / All	p. 16 (Conflict of Interest Statement)
	**14c**	Indicate where the study protocol can be accessed or state that a protocol was not prepared.	H / All	NA — Not Required
	**14d**	Provide registration information, or state that the study was not registered.	H / All	NA — Not Required
	**14e**	Provide details of the availability of the study data.	All / All	p. 16 (Data Availability Statement); Data S1; Appendix S1
	**14f**	Provide details of the availability of the code to reproduce the study results.	All / All	p. 16 (Data Availability Statement — analysis code is not publicly deposited; the full analytical protocol, statistical specifications and all input data required to reproduce the results are provided in Appendix S1 and Data S1)
**Public Involvement**	**15**	Provide details of any patient and public involvement or state no involvement.	H / All	NA — Not Required
**Results**				
**Participants**	**16a**	When using patient/EHR data, describe the flow of text/EHR/patient data through the study.	E,H / All	NA — no patient or EHR data. Flow of the analyzed item set: p. 4 (Figure 1); p. 5 (Table 1)
	**16b**	When using patient/EHR data, report characteristics overall and by source/setting and development/evaluation split.	E,H / All	NA — no patient or EHR data. Item characteristics by domain and consensus level: p. 5 (Table 1)
	**16c**	For LLM evaluation, compare the distribution of important predictors between development and evaluation data.	E,H / All	NA — evaluation-only design; no development dataset exists for comparison
	**16d**	When using patient/EHR data, specify the number of participants and outcome events in each analysis.	E,H / All	NA — no patient or EHR data. Number of items in each analysis: p. 5 (Table 1, n = 166); p. 8 (Table 3, hierarchy strata); pp. 9–10 (Tables 4–5, n = 40 subset); p. 11 (Table 6)
**Performance**	**17**	Report LLM performance according to pre-specified metrics and/or human evaluation.	All / All	pp. 7–8 (Table 2); p. 8 (Table 3); pp. 8–9 (response distribution and ceiling effect); pp. 9–10 (Tables 4–5); pp. 10–11 (subgroup, theme-level and prior-exposure results, Table 6)
**LLM Updating**	**18**	If applicable, report the results from any LLM updating and subsequent performance.	All / All	NA — no model updating was performed; p. 14 notes that findings characterize the systems as deployed in January 2026
**Discussion**				
**Interpretation**	**19a**	Give an overall interpretation of the main results, including issues of fairness, in the context of the objectives and previous studies.	All / All	p. 11 (Discussion, opening); p. 12 (What the reference standard represents — panel geography and specialty composition); pp. 12–14 (rank-order alignment, response bias, domain heterogeneity, inter-model differences)
**Limitations**	**19b**	Discuss any limitations of the study and their effects on biases, statistical uncertainty, and generalizability.	All / All	p. 15 (Limitations, items 1–8)
**Usability of the LLM in context**	**19c**	Describe known challenges in using data for the specified task and domain with reference to representation, missingness, harmonization, and bias.	E,H / All	p. 12 (representation and panel composition bias); p. 13 (positive response bias and elicitation format); pp. 13–14 (under-representation of technical detail in the literature); p. 15 (Limitations 1, 3, 4, 6)
	**19d**	Define the intended use for the implementation under evaluation, including intended input, end-user, and level of autonomy/human oversight.	E,H / All	p. 3 (Introduction, final paragraph — models characterized as representations of expert opinion, not substitutes for expert judgement); pp. 15–16 (Conclusion — orientation aid for investigators under human oversight; not a substitute for formal consensus processes)
	**19e**	If applicable, describe how poor quality or unavailable input data should be assessed and handled when implementing the LLM.	E,H / All	p. 13 (dependence of measured bias on elicitation format); p. 15 (Limitations 2 and 4 — single-query elicitation, response instability, format effects)
	**19f**	If applicable, specify whether users will be required to interact in the handling of input data or use of the	E,H / All	pp. 15–16 (Conclusion — outputs require expert appraisal; models cannot substitute for formal

		LLM, and the level of expertise required.		consensus where the endorsed/contested distinction matters)
	**19g**	Discuss next steps for future research, with a view to applicability and generalizability of the LLM.	All / All	p. 14 (need for longitudinal reassessment as models are revised); p. 15 (Limitation 8 — retrieval augmentation, chain-of-thought prompting, fine-tuning, other systems)
