# Supplementary material for: Alignment Between Large Language Models and Consensus in Endoscopic Spinal Disc Surgery: A Comparative Analysis Against the Neurocore‐SENSED
Source: JOR Spine. 2026 Sep 4;9(3):e70216. doi: 10.1002/jsp2.70216 (PMC13545091; doi:10.1002/jsp2.70216)
Supplement: Supplementary file 2 [file JSP2-9-e70216-s001.docx]

**Supplementary Tables**

*Alignment Between Large Language Models and Consensus in Endoscopic Spinal Disc Surgery: A Comparative Analysis Against the Neurocore-SENSED*

**Supplementary Table S1. Cross-tabulation of consensus level against collapsed model response (n = 166 items).**

| **Model** | **Consensus level** | **Model response 1–2** | **Model response 3** | **Model response 4–5** | **Row total** |
| --- | --- | --- | --- | --- | --- |
| Claude Sonnet 4.6 | Strong (≥75%) | 0 | 2 | 80 | 82 |
|  | Moderate (66–74%) | 0 | 2 | 14 | 16 |
|  | No consensus (<66%) | 2 | 25 | 41 | 68 |
| Gemini 3 Pro | Strong (≥75%) | 0 | 0 | 82 | 82 |
|  | Moderate (66–74%) | 0 | 1 | 15 | 16 |
|  | No consensus (<66%) | 2 | 18 | 48 | 68 |
| GPT-5.2 | Strong (≥75%) | 0 | 0 | 82 | 82 |
|  | Moderate (66–74%) | 0 | 0 | 16 | 16 |
|  | No consensus (<66%) | 4 | 3 | 61 | 68 |

*Model responses were collapsed as 1–2 (disagreement), 3 (undecided), and 4–5 (agreement) to match the three consensus levels. Cells on the leading diagonal represent exact agreement. All three models placed the majority of no-consensus items in the agreement category, which is the source of both the limited observed agreement and the marginal imbalance that renders weighted kappa uninterpretable as a comparative measure (see main manuscript, Table 2).*

**Supplementary Table S2. Items showing the greatest discordance between panel endorsement and model response.**

| **Item** | **Theme** | **Panel (%)** | **Claude Sonnet 4.6** | **Gemini 3 Pro** | **GPT-5.2** |
| --- | --- | --- | --- | --- | --- |
| **Panel endorsement below 50%, all models in agreement range (n = 31)** |  |  |  |  |  |
| Modified Macnab Criteria | Clinical severity scoring | 21 | 4 | 4 | 4 |
| Antibiotics | Active treatments | 22 | 4 | 4 | 5 |
| Neurological - reflexes | Outcome indicators | 23 | 4 | 4 | 4 |
| Functional - modified Macnab criteria | Outcome indicators | 24 | 4 | 4 | 4 |
| Imaging resolution (CT or MRI) | Outcome indicators | 28 | 4 | 4 | 5 |
| Use of forceps/graspers | Technical practice | 30 | 4 | 4 | 4 |
| mJOA scoring system | Clinical severity scoring | 30 | 4 | 4 | 4 |
| Saddle anesthesia or bowel/bladder dysfunction | Clinical severity scoring | 30 | 5 | 5 | 5 |
| Reflex changes | Clinical severity scoring | 31 | 4 | 4 | 4 |
| operating table type (e.g. radiolucent) | Surgical practice | 32 | 4 | 4 | 4 |
| Performing electrophysiological studies | Clinical practice | 33 | 4 | 4 | 4 |
| Night pain | Initial complaint | 36 | 4 | 4 | 4 |
| Functional - SF-36 | Outcome indicators | 37 | 4 | 4 | 4 |
| NRS pain scoring system | Clinical severity scoring | 38 | 5 | 5 | 5 |
| Pfirrmann grading system | Morphological severity | 38 | 4 | 4 | 5 |
| pain management programs (multidisciplinary approach) | Clinical practice | 39 | 4 | 4 | 5 |
| Dermatomal sensory loss distribution | Clinical severity scoring | 39 | 5 | 5 | 5 |
| Provocative tests (e.g. Straight Leg Raise, Spurling's) | Clinical severity scoring | 41 | 5 | 5 | 5 |
| MRC weakness severity scoring system (grade ≤3/5) | Clinical severity scoring | 41 | 5 | 5 | 5 |
| Functional - return to sports or high-impact activities (days) | Outcome indicators | 41 | 4 | 4 | 4 |
| Closure technique | Surgical practice | 42 | 4 | 4 | 5 |
| Instrument failure or breakage | Complications | 42 | 5 | 5 | 5 |
| Alcohol consumption | Demographics | 42 | 4 | 4 | 4 |
| specific devices (e.g. Wilson frame) | Surgical practice | 43 | 4 | 4 | 4 |
| Use of cannulas and tubes | Technical practice | 43 | 4 | 4 | 4 |
| lifestyle modifications | Clinical practice | 45 | 4 | 4 | 4 |
| Active malignancies | Comorbidities | 45 | 5 | 5 | 5 |
| Familiar history of disc herniation | Demographics | 45 | 4 | 4 | 5 |
| Injury to major structures (e.g. esophagus, trachea, lung) | Complications | 48 | 5 | 5 | 5 |
| Size of skin incision(s) | Surgical practice | 48 | 4 | 4 | 5 |
| Neurological - sensory | Outcome indicators | 49 | 5 | 5 | 5 |
| **Panel endorsement 85% or above, at least one model not in agreement range (n = 1)** |  |  |  |  |  |
| pump type and settings (e.g., flow rate, pressure) | Technical practice | 92 | 3 | 4 | 5 |

*The upper block lists every item endorsed by fewer than 50% of the panel to which all three models responded in the agreement range (4 or 5). The lower block lists every item endorsed by at least 85% of the panel to which at least one model did not respond in the agreement range. The asymmetry between the two blocks — 31 items against one — indicates that discordance is almost entirely unidirectional, with models over-endorsing rather than under-endorsing relative to the expert panel.*
