# Supplementary material for: Alignment Between Large Language Models and Consensus in Endoscopic Spinal Disc Surgery: A Comparative Analysis Against the Neurocore‐SENSED
Source: JOR Spine. 2026 Sep 4;9(3):e70216. doi: 10.1002/jsp2.70216 (PMC13545091; doi:10.1002/jsp2.70216)
Supplement: Supplementary file 3 [file JSP2-9-e70216-s003.docx]

**Supplementary Methods**

*Alignment Between Large Language Models and Consensus in Endoscopic Spinal Disc Surgery: A Comparative Analysis Against the Neurocore-SENSED*

**S1.1 Prompts and query conditions**

Models and access. Three commercially available general-purpose large language models were queried: OpenAI GPT-5.2, Google Gemini 3 Pro, and Anthropic Claude Sonnet 4.6. All queries were administered in January 2026 through each provider’s standard paid-tier consumer conversational interface. Consumer interfaces do not expose temperature, top-p, or related inference parameters; these were therefore left at provider defaults and cannot be reported as specific values. No system prompt, custom instruction, memory feature, or personalization setting was applied, and web retrieval was disabled in each interface.

Session handling. Each of the 166 items was submitted once per model. Every query was issued in a new conversation containing no prior context. No follow-up turns were used, no message was edited and resubmitted, and no item was queried in the same session as another item.

Prompt. The following prompt was used verbatim for every item. The item text replaced the final clause; where an item was nested beneath a parent item in the source questionnaire, the parent item text preceded the sub-item so that the model received the same context as the Delphi panelists.

You are an experienced spine surgeon with expertise in endoscopic spine surgery. Evaluate the following statement according to your current medical knowledge and clinical judgment only. Do not attempt to recall or reproduce any published guideline or consensus document, and do not use external resources. In endoscopic spine surgery for disc disease, should the following be systematically reported in research? Rate your level of agreement using exactly one of the options: Strongly disagree, Disagree, Undecided, Agree, or Strongly agree.

Response capture. The prompt constrains the model to emit one of five ordered category labels. Labels were transcribed to their ordinal values (strongly disagree = 1 through strongly agree = 5) without interpretation. No free-text reasoning was elicited, and no human rating, coding, or adjudication occurred at any stage. Both the emitted label and its numeric value are reported for every item in Data S1.

Role framing. The prompt assigns the model the role of an experienced endoscopic spine surgeon. This was chosen so that the elicited judgement would be comparable in framing to that of the Delphi panelists. Responses obtained without role framing may differ, and this is acknowledged as a limitation.

**S1.2 Sensitivity analyses**

Score-to-category mapping. The primary analysis collapsed the five response options into three categories (1–2, 3, 4–5) to permit comparison with the three-level consensus classification. Because this rule is analytically arbitrary, the categorical statistics were recomputed under two alternative mappings: 1–3 / 4 / 5, and 1–2 / 3–4 / 5. Results are reported in Table 3 of the main manuscript.

Item hierarchy. The source item bank nests certain sub-items beneath parent items, in some cases as mutually exclusive alternatives (for example, operative duration reported in minutes, hours, or seconds). Of the 166 analyzed items, 128 were parent items and 38 were nested sub-items. Spearman correlations were recomputed separately within each stratum to establish whether the primary finding depended on item structure.

Exposure-implicated items. One item was correctly reproduced by one model during the contamination probe (Supplementary S1.3). The primary correlation was recomputed with that item removed, for each model, to quantify any contribution of prior exposure to the reported association.

Uncertainty estimation. Confidence intervals for all correlation coefficients and agreement statistics were obtained by bootstrap resampling of items, using the percentile method with 5000 resamples and a fixed random seed. Correlations between models, which share panel endorsement as a common variable, were compared using the Williams modification of the Steiger test for dependent correlations. Theme-level analyses were corrected for multiplicity by the Benjamini–Hochberg procedure across all evaluable tests.

**S1.3 Protocols addressing measurement reproducibility**

**Protocol 1 — Response stability under repeated sampling**

A subset of 40 items was drawn by stratified random sampling across consensus levels (16 no consensus, 4 moderate, 20 strong), using a fixed random seed. Each item was submitted three times to each model, giving 360 queries, every query issued in a new session with no prior context and under the conditions described in S1.1. Agreement across the three runs was quantified by Krippendorff’s alpha for ordinal data, together with the proportion of items returning an identical score on all three runs and the proportion whose scores spanned no more than one scale point. The primary correlation was additionally recomputed separately for each run within the subset.

**Protocol 2 — Effect of response format**

The same 40 items were resubmitted once per model using a forced-choice prompt requiring a bare digit as output. The prompt was:

You are an experienced spine surgeon with expertise in endoscopic spine surgery. Evaluate the following statement according to your current medical knowledge and clinical judgment only. Do not attempt to recall or reproduce any published guideline or consensus document, and do not use external resources. If uncertain, select the option that best reflects your current opinion rather than refusing to answer. In endoscopic spine surgery for disc disease, should the following be systematically reported in research? Answer with a single digit only, using this scale: 1 = strongly disagree, 2 = disagree, 3 = undecided, 4 = agree, 5 = strongly agree. Output the digit and nothing else.

Paired item-level scores were compared with the original responses by Wilcoxon signed-rank test, and the proportion of responses in the top two categories was compared between conditions. Two features distinguish this prompt from the original: the response format, and an added instruction not to decline to answer. Their contributions cannot be separated in this design, and this is stated as a limitation in the main manuscript.

**Protocol 3 — Prior exposure to the reference consensus**

The reference consensus was published online on 13 September 2025 under a CC-BY license, before the January 2026 query window, so prior exposure was possible in principle for all three models. Ten fixed probes were administered once per model, each in a new session with web retrieval disabled. Probes P01–P04 requested publication details and methodological parameters; P05–P07 requested item-level endorsement percentages that appear only in the source appendices; P08 and P09 named items that do not exist in the framework and served as controls, since a confident answer to either demonstrates confabulation and recalibrates how an apparently accurate answer to P05–P07 should be read; P10 verified that no external source had been consulted. Responses were classified as no recall, partial, accurate recall, or confabulated. Verbatim probe wording and responses are provided in Data S1; the classification is summarized in Table 6 of the main manuscript.

**S1.4 Software**

All analyses were performed in Python 3.11 using SciPy 1.11, NumPy, and pandas. Gwet’s AC1 and AC2 and the prevalence-adjusted bias-adjusted kappa were implemented directly from their published definitions, as was the Williams modification of the Steiger test.
